# Electrodeposited Carbonyl Functional Polymers as Suitable Supports for Preparation of the First-Generation Biosensors

**DOI:** 10.3390/s23073724

**Published:** 2023-04-04

**Authors:** Milan Sýs, Michaela Bártová, Tomáš Mikysek, Ivan Švancara

**Affiliations:** Department of Analytical Chemistry, Faculty of Chemical Technology, University of Pardubice, Studentská 573, 532 10 Pardubice, Czech Republic

**Keywords:** carbonyl functional polymer, electropolymerization, Schiff base formation, amperometric detection, enzyme, catalytic biosensor, phenolic compounds

## Abstract

The aim of this electrochemical study was to ascertain which type of electrochemically deposited carbonyl functionalized polymer represents the most suitable electrode substrate for direct covalent immobilization of biological catalysts (enzymes). For this purpose, a triad of amperometric biosensors differing in the type of conductive polymers (poly-vanillin, poly-*trans*-cinnamaldehyde, and poly-4-hydroxybenzaldehyde) and in the functioning of selected enzymes (tyrosinase and alkaline phosphatase) has been compared for the biosensing of neurotransmitters (dopamine, epinephrine, norepinephrine, and serotonin) and phenyl phosphates (*p*-aminophenyl phosphate and hydroquinone diphosphate). The individual layers of the polymers were electrochemically deposited onto commercially available screen-printed carbon electrodes (type C110) using repetitive potential cycling in the linear voltammetric mode. Their characterization was subsequently performed by SEM imaging and attenuated total reflectance FTIR spectroscopy. Molecules of enzymes were covalently bonded to the free carbonyl groups in polymers via the Schiff base formation, in some cases even with the use of special cross-linkers. The as-prepared biosensors have been examined using cyclic voltammetry and amperometric detection. In this way, the role of the carbonyl groups embedded in the polymeric structure was defined with respect to the efficiency of binding enzymes, and consequently, via the final (electro)analytical performance.

## 1. Introduction

Generally, enzyme immobilization via a covalent bonding to an insoluble conductive polymer electrodeposited onto the surface of electrochemical transducers (electrodes) guarantees high mechanical stability and reproducible serial production, in contrast to the physical anchoring of enzyme molecules (via so-called encapsulation) into the net structure of a polymer, without need to engage functional groups that mediate the covalent chemical bonding [1]. Hence, it can be assumed that it is only a matter of time until, for example, electrodeposited amino-functional polymers such as polytyramine [2], polyhistamine [3], and polyarginine [4] will find their use in enzyme immobilization, like the already commonly used chitosan [5], poly(ethylene glycol) diglycidyl ether (PEGDE) [6], poly(ethylene glycol) diamine, polyallylamine, or poly-l-arginine, which are substrates applicable using a simple drop-casting method [7].

Here, it is necessary to emphasize that a physical method of immobilization may block the access of the analyte to the active center of an enzyme, which often results in a decrease in the sensitivity of such catalytic sensors. Despite their disadvantages, the second type of electrodeposited polymers which includes polyaniline (PANI) [8], polypyrrole (PPy) [9], poly-3,4-ethylenedioxythiophene (PEDOT) [10], and copolymers derived from (see [11]), is still preferred in the construction of biosensors; this is perhaps due to their easier electropolymerization.

In studies on the electrochemical behavior of phenolic substances, the respective authors are often confronted with the so-called passivation of the working electrode surface after anodic oxidation of these substances [12]. This is manifested in an increase in the capacitive current (*I*_C_, see [13]) or, more generally, in enhanced background currents [12]. Moreover, the products formed (typically intermediates) undergo subsequent chemical reactions that are either followed by further electrochemical transformation, or they participate in polymerization. The corresponding reaction mechanisms have already been utilized for the preparation of polymer layers, thus serving as components of microbial fuel cells [13], amine-oxidase-mimicking sustainable catalysts (polydopamine) [14], corrosion protectors (polytyramine) [15], preservatives with antioxidant and antimicrobial activity (polyeugenol) [16]; they may also act as components in the development of highly sophisticated sensors applicable to polycurcumin [17], or as a tumor inhibitor [18], etc.

In their molecular structures, some phenolic monomers subjected to electropolymerization usually contain specific functional groups that can be involved in reactions with biological catalysts (through covalent enzyme immobilization) acting in more sophisticated biorecognition layers [19] that are applicable in the development of specific analytical devices [20]. These functional groups include primary amino groups (polytyramine) [21], carboxylic groups (poly-*p*-coumaric acid) [22], etc. Thus, there is an assumption that it may be possible to prepare also polymers containing the carbonyl (R-CH=O) or formyl groups (Ar-CH=O) in an analogous way. In addition, it may be possible to link these carbonyl groups directly with enzyme molecules (free amino groups from basic amino acids) via simple Schiff base formation, which is the central theme of this report.

In comparison, completely new conductive polymers with carbonyl and formyl groups can exhibit distinct catalytic activity and can significantly increase the electrochemically active surface area of the corresponding transducers, even without the presence of commonly preferred new kinds of carbon (i.e., graphene sheets, nanotubes, nanohorns or fullerenes), in which the covalent immobilization via Schiff base formation [23] enables the use of all the active sites of the total number of bounded enzymes, as demonstrated in the study reported herein.

In this regard, it was necessary to design procedures for the electrodeposition of three different conductive polymeric supports, namely poly-vanillin [24], poly-*trans*-cinnamaldehyde [25], and poly-4-hydroxybenzaldehyde [26], onto a commercially available screen-printed carbon electrode (SPCE). The selection of the individual monomers was intentional and their polymerization performed with the aid of repetitive potential cycling in the linear voltammetric mode. If molecular structures of *trans*-cinnamaldehyde and 4-hydroxybenzaldehyde are compared, it should be evident that the missing hydroxy group on the benzene ring can have a significant effect on the electropolymerization. Furthermore, in the case of vanillin, the presence of an electron-donating methoxy group can also affect the formation of the corresponding poly-vanillin. From their comparison, it may be possible to derive suitable phenolic monomers. The resulting polymers with bonded enzymes (tyrosinase and alkaline phosphatase) have been characterized in detail and tested for electroanalytical purposes, especially in amperometric biosensing of selected catecholamines (acting as neurotransmitters) [27] and phenyl phosphates (preferred as specific immunoassay reagents [28]). Their potential employment as a new type of specific detector incorporated in the wall-jet flow cell is discussed, experimentally demonstrated, and critically assessed. Moreover, amperograms measured in the hydrodynamic arrangement could serve as a convenient tool for checking the stability of bound enzyme molecules.

## 2. Materials and Methods

### 2.1. Chemicals and Reagents

The following chemicals, substances, and solvents purchased from Merck KGaA (Darmstadt, Germany) were used: vanillin (≥97%), *trans*-cinnamaldehyde (≥99%), 4-hydroxybenzaldehyde (≥95.0%), buffered aqueous glycerol solution of alkaline phosphatase (EC 3.1.3.1) from bovine intestinal mucosa (7078 U mg^−1^) with a storage temperature of 2–8 °C, lyophilized powder of tyrosinase (EC 1.14.18.1) from mushroom (8503 U mg^−1^) with a storage temperature of −20 °C, *p*-aminophenyl phosphate disodium salt monohydrate (pAPP), hydroquinone diphosphate (HQDP), *p*-aminophenol, hydroquinone, glutaraldehyde (GTA) solution (50% *w*/*w* in H_2_O), dopamine hydrochloride (USP reference standard), (±)-epinephrine hydrochloride, DL-norepinephrine hydrochloride (≥98%) serotonin hydrochloride, lithium perchlorate (99.99% trace metals basis), acetonitrile for HPLC (≥99.9%) and ethanol (96%). Furthermore, the inorganic components needed for the preparation of working media also included glacial acetic acid (USP reference standard), *o*-phosphoric acid (85%), sulfuric acid (95%), boric acid (≥99.5%) sodium hydroxide, sodium phosphate dibasic dihydrate, sodium dihydrogen phosphate monohydrate, and sodium acetate anhydrous (≥99.0%), all being obtained from Lach-Ner, s.r.o. (Neratovice, Czech Republic). Highly purified water of resistivity 18 MΩ cm was prepared by a Milli-Q^®^ deionization unit from Merck Millipore (Burlington, MA, USA).

### 2.2. Apparatus and Other Accessories

Three different carbonyl functional polymers were electrodeposited using repetitive potential cycling (RPC or, alternatively, repetitive cyclic voltammetry) in a conventional three-electrode arrangement consisting of a commercially available SPCE (type “DRP-C110”) from Metrohm DropSens (Oviedo, Spain) that had served as the working electrode. A silver/silver chloride (Ag/AgCl) electrode with 3 mol L^−1^ KCl for aqueous media and a saturated calomel electrode (SCE) with 0.1 mol L^−1^ LiClO_4_ in pure acetonitrile (MeCN) as a salt bridge for nonaqueous media represented the reference electrodes (both Metrohm, s.r.o.; Prague, Czech Republic), and a platinum plate (Elektrochemické detektory, s. r. o.; Turnov, Czech Republic) played the role of the auxiliary electrode.

Using a VEGA3 SBU instrument from TESCAN s.r.o. (Brno, Czech Republic), imaging by scanning electron microscopy (SEM) and energy dispersive X-ray analysis (EDX) were carried out to map the microstructure of the polymeric layers and surface distribution of the covalently bonded enzyme molecules, respectively. To achieve greater magnification (6.64 × 10^3^), the electrodes were coated with a 5 nm layer of pure gold, using a rotary pumped coater (type Q150R Plus) from Quorum Technologies (Lewes, UK). To identify and confirm the presence of carbonyl groups in electrochemically deposited polymers, attenuated total reflectance Fourier transform infrared spectroscopy (ATR-FTIR) of polymeric layers had to be carried out using a Nicolet™ iS50 FTIR Spectrometer from Thermo Fisher Scientific™ (Waltham, MA, USA).

All amperometric experiments were performed with the aid of an FIA setup incorporating a multichannel peristaltic pump MINIPULS 3 from Gilson, Inc. (Middleton, WI, USA), a Rheodyne^®^ automatic six-stage dosing valve from IDEX Health & Science GmbH (Wertheim, Germany), a wall-jet flow cell from Metrohm DropSens (Oviedo, Spain) and a computer with operational software NOVA 1.11, enabling us to control a potentiostat/galvanostat AUTOLAB model PGSTAT101 connected to the SPCE bearing covalently immobilized enzymes.

### 2.3. Procedures

#### 2.3.1. Preparation of Carbonyl Group Functionalized Polymers

The poly-vanillin modified SPCE was prepared using a solution of 2 mg mL^−1^ vanillin in 0.1 mol L^−1^ PB (pH 7) in an electrochemical cell; this was achieved by applying the potential varied from −0.8 to +1.8 V at a scan rate of 200 mV s^−1^ for multiple (50) cycles. The poly-*trans*-cinnamaldehyde was electrochemically deposited onto the bare SPCE using repetitive potential cycling in a medium of 10 mL 96% EtOH, 0.5 mol L^−1^ H_2_SO_4_ (7:3 in *v*/*v*) and 20 mg *trans*-cinnamaldehyde in the range of −0.6 to +2.0 V at a scan rate of 200 mV s^−1^, when applying, in total, 50 consecutive cycles. Finally, the poly-4-hydroxybenzaldehyde electrodeposited onto SPCE was prepared analogically by cycling in a solution of in 0.1 mol L^−1^ PB (pH 7) with 2 mg mL^−1^ 4-hydroxybenzaldehyde, in the range of −0.6 to +2.0 V at a scan rate of 200 mV s^−1^, also for 50 cycles.

#### 2.3.2. Immobilization of Alkaline Phosphatase on Polymeric Supports

To prepare the stock solution of alkaline phosphatase (ALP), 2 μL of buffered aqueous glycerol solution of alkaline phosphatase was added to 48 μL of 0.1 mol L^−1^ PB with 0.1 mol L^−1^ KCl. After mixing, 10 μL of the resulting stock solution of ALP was drop-casted onto the surface of the bare SPCE and its modifications. Then, pure distilled water had to be dripped permanently to prevent drying of the electrode surface. The formation of Schiff bases between the polymeric supports and the ALP molecules was allowed to proceed minimally for 2 h, which was necessary for the individual ALP molecules to cross-link using 2.5 μL 1% GTA when the analogical procedure with distilled water was repeated. After drying, the surfaces of the resulting biosensors were rinsed with water and stored in a refrigerator at 5 °C.

#### 2.3.3. Immobilization of Mushroom Tyrosinase on Polymeric Supports

In an Eppendorf^®^ plastic tube, a stock solution of 2 mg mL^−1^ tyrosinase (TYR) was prepared by dissolving the appropriate amount in 2 mL of 0.1 mol L^−1^ PB with 0.1 mol L^−1^ KCl content. A volume of 5 μL TYR stock solution was drop-casted onto the surface of bare SPCE and its modifications. Then, the same procedure as for ALP immobilization was used.

#### 2.3.4. Amperometric Detection in Batch Configuration

Amperometric measurements in batch configuration were chosen to compare the functioning of the ALP biosensors. Regarding optimal ALP biocatalytic activity and the use of a silver plate pseudo-reference electrode, all the experiments were performed in 0.1 mol L^−1^ carbonate-bicarbonate buffer (CBB) with 0.1 mol L^−1^ KCl (pH 9). If not stated otherwise, a working potential of −0.1 V and a stirring speed of 400 rpm were the values of choice.

#### 2.3.5. Amperometric Detection in a Flow-Injection System

The wall-jet configuration with a pseudo-reference silver electrode was used in combination with TYR amperometric biosensor(s) and a supporting electrolyte of 0.1 mol L^−1^ phosphate buffer +0.1 mol L^−1^ KCl (pH 7.0, non-de-aerated solution). This choice for FIA experiments was due to optimal TYR biocatalytic activity at a neutral pH. Amperometric detection was usually performed at −0.2 V vs. ref. at a flow rate of 1.0 mL min^−1^. Before each analysis, the flow cell with a fresh biosensor had to be rinsed with water for at least 5 min in order to split out the adsorbed (physically immobilized) enzyme from the polymer support, which ensured the constant current signal. Otherwise, the data given above do not always represent the ultimate set-up of working conditions and instrumental parameters for maximal analytical performance under test. Each change in the working conditions listed above is given in the corresponding figure(s).

## 3. Results and Discussion

### 3.1. Electropolymerization of Carbonyl Functional Monomers

**Figure 1:**
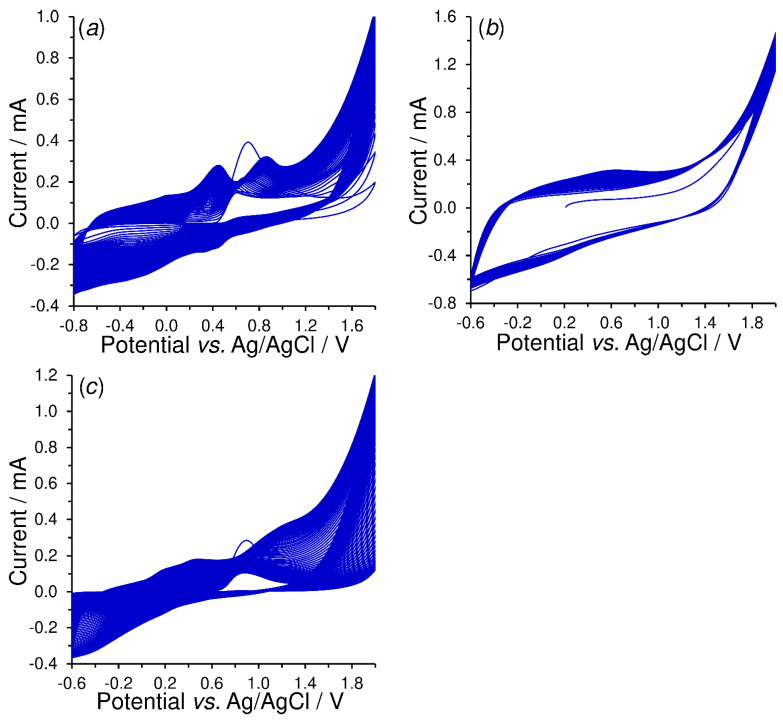
A set of voltammograms obtained by repetitive potential cycling (RPC, *n* = 50) of 2 mg mL^−1^ vanillin in 0.1 mol L^−1^ PB of pH 7 (**a**), 2 mg mL^−1^ cinnamaldehyde dissolved in mixture of 0.5 mol L^−1^ H_2_SO_4_ and 96% ethanol (3:7) (**b**), and 2 mg mL^−1^ 4-hydroxybenzaldehyde in 0.1 mol L^−1^ PB of pH 7 (**c**) recorded on SPCE at *E*_step_ = 5 mV and *ν* = 200 mV s^−1^.

Electropolymerization of vanillin, cinnamaldehyde, and 4-hydroxybenzaldehyde was carried using RCV (see Figure 1) to form the corresponding conductive polymers that would contain carbonyl (poly-*trans*-cinnamaldehyde) formyl groups (poly-vanillin and poly-4-hydroxybenzaldehyde) suitable for covalent binding of enzymes without the need to use special linkers. Although there already exist some instructions for their laboratory preparation [24,25,26], the individual electropolymerization parameters, namely, the composition of the polymerization media, potential range, scan rate, and the number of cycles, had to be optimized.

The polymer formation is indicated by the increase in background currents (here mainly capacitive current) after each cycling due to the electrode surface being blocked by a less conductive polymer. As found experimentally, electropolymerization of vanillin and 4-hydroxybenzaldehyde can be recommended for neutral aqueous solutions unlike poorly water soluble cinnamaldehyde, whose transformation to polymer had to be carried out in acidified alcohol medium. The working conditions of RPC had included scanning towards extremely high potentials (up to +2 V vs. ref.), as the available anodic window (reversely to 0 V) was not sufficient; however, the respective experiment had to be continued down to −0.6 V. Moreover, the setting of lower scan rates (from 5 to 50 mV s^−1^) did not allow us to attain the full coverage of the working surface. Scanning at 200 mVs^−1^ with a minimum of 50 cycles can be considered optimal, because it has been possible to reach an average value of background currents of about 5 μA in pure 0.1 mol L^−1^ acetate buffer (AcB, pH 4.5).

**Figure 2:**
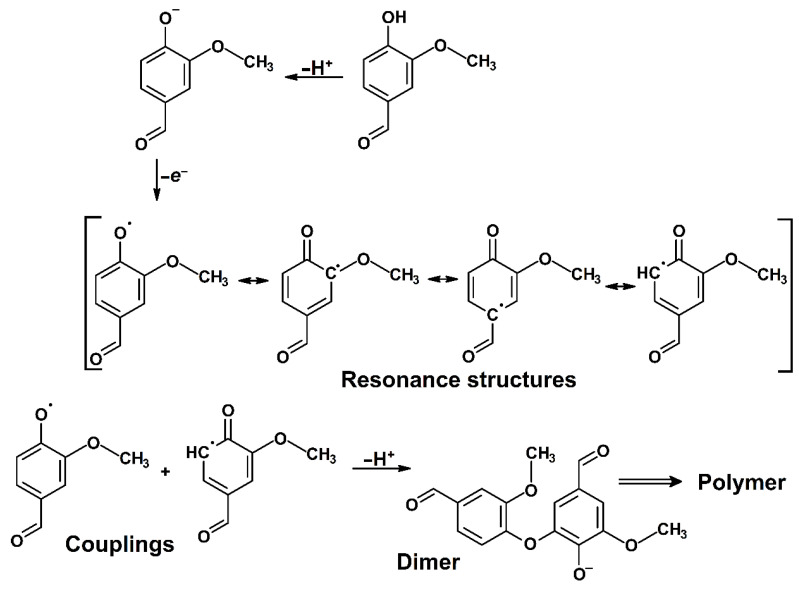
Proposed reaction mechanism of electropolymerization of vanillin.

Usually, it is not possible to propose the exact chemical structure of electrodeposited polymers, since the electrochemical behavior of selected monomers is controlled mainly by the electron transfer at the electrode surface with the respective pathway, in this case via the ECE mechanism (this is demonstrated in Scheme S1, included in the Supplementary Materials). As a consequence of slower hydroxylation reaction, which is anodic oxidation of monomer taking place via the two one-electron processes plus one proton and is completed by the subsequent nucleophilic addition of water, one can expect a coupling reaction between the radicals of monomers formed electrochemically by consuming one electron and proton (see Figure 2).

**Figure 3:**
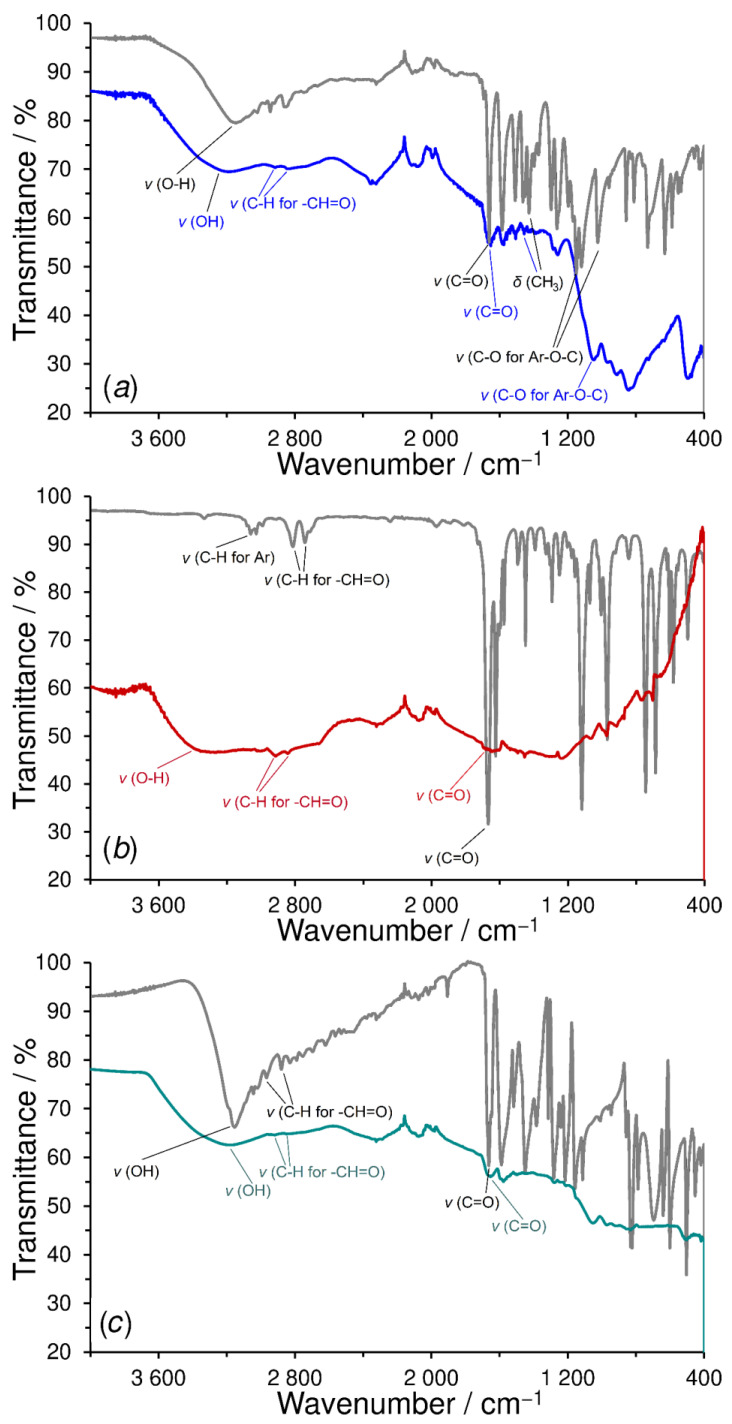
FTIR spectra of vanillin with poly-vanillin (**a**), *trans*-cinnamaldehyde with poly-*trans*-cinnamaldehyde (**b**), and 4-hydroxybenzaldehyde with poly-4-hydroxybenzaldehyde (**c**). Spectra of monomers are colored in gray.

As demonstrated in Figure 3, the assumption given above was confirmed by comparing the ATR-FTIR spectra of monomers with the corresponding polymers prepared by electrodeposition. The presence of typical bands of the valence vibrations for aryl ethers ν (C-O) at 1050 cm^−1^ and bands of deformation vibrations δ (CH_3_) of ~1460 cm^−1^ in the poly-vanillin IR spectrum (see Figure 3a) corresponds to the fact that during potential cycling (electropolymerization), the electrode reaction pathway of vanillin in Scheme S1 does not dominate, hence 100% detachment of the molecule of methanol does not occur.

Furthermore, the bands belonging to ν (C-H) stretching in the aromatic ring at ~3000–3080 cm^−1^ could not be observed in the IR spectra of all three polymers due to the presence of hydroxyl groups (in water). Nevertheless, adsorption bands for ν (C-H) of the carbonyl group at ~2850–2940 cm^−1^ and ν (C=O) of the carbonyl group at ~1660 cm^−1^ were evident enough in the poly-vanillin and poly-4-hydroxybenzaldehyde spectra (see Figure 3a,c), indicating the presence of carbonyl groups involved in the subsequent covalent binding of biocatalysts.

In the case of the poly-*trans*-cinnamaldehyde IR spectrum (see Figure 3b), the absorption bands that would have indicated the presence of carbonyl groups were difficult to recognize, probably due to a thinner polymer layer. This statement can be deduced from the electrochemical behavior of *trans*-cinnamaldehyde, which does not contain a phenolic group (or thiol) in its structure (in *para* or *ortho* position) that had to be first electrochemically synthesized (via the hydroxylation reaction of benzene ring). To do so by means of nucleophilic addition of a hydroxyl group, the cycling of 2 mg mL^−1^ *trans*-cinnamaldehyde in a mixture of 0.1 mol L^−1^ carbonate-bicarbonate buffer (CBB) +0.1 mol L^−1^ KCl (pH 9) in 96% ethanol (7:3) was also tried, unfortunately without significant improvement.

Hence, it can be concluded here that substances such as *p*-coumaraldehyde, 3-(4-hydroxy-phenyl)-propionaldehyde, and their thiolic analogues represent more suitable monomers for the preparation of carbonyl functional polymers.

### 3.2. Microscopic Analysis of Carbonyl Functional Polymers

**Figure 4:**
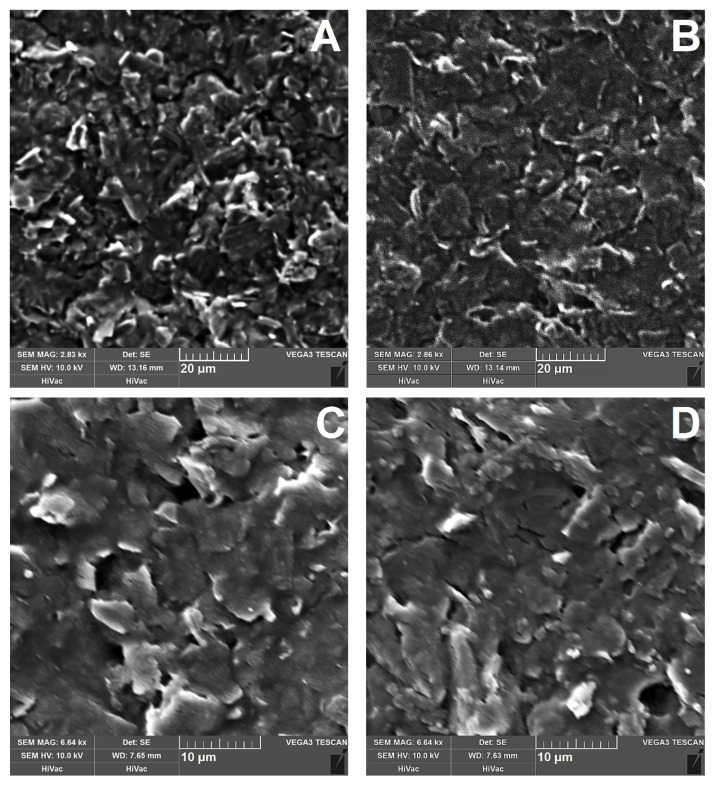
Typical SEM images (~2.85 × 10^3^ magnification) of bare SPCE (type DRP-C110; (**A**)), SCPE covered with thin layer of poly-vanillin (**B**), SEM images (~6.64 × 10^3^ magnification) of SPCE/PV-ALP (**C**) with SPCE/P4HB-TYR (**D**).

Scanning electron microscopy (SEM) utilizing the secondary electrons (SE) imaging mode was used for mapping the microstructures of bare SPCE (type DRP-C110) and three different polymeric layers, namely poly-vanillin (SPCE/PV), poly-*trans*-cinnamaldehyde (SPCE/PC), and poly-4-hydroxybenzaldehyde (SPCE/P4HB). As evident from the comparison of images in Figure 4, the surface of the bare SPCE consists of graphite sheets connected with a thin layer of binder (A), while the dark coating of poly-vanillin on the surface of the conductive graphite sheets indicates the presence of the polymer (B). The presence of the polymer is much more evident in images C and D that illustrate the morphology of surfaces with covalently bound enzymes. It seems that the resulting biorecognition layers exhibit a compact (tightly bound) structure.

**Figure 5:**
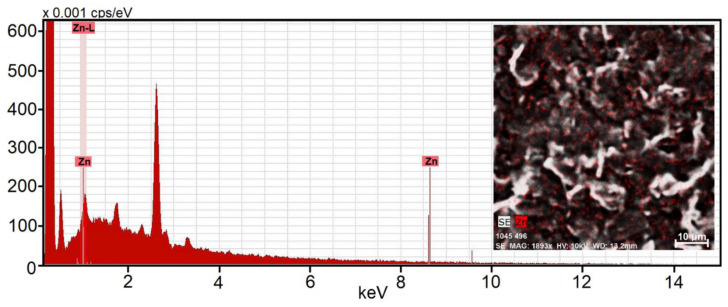
Energy dispersive X-ray spectrum of the screen-printed carbon electrode (type DRP-C110) covered with a thin layer of poly-4-hydroxybenzaldehyde and covalently bound ALP. The inset image shows a map of zinc’s distribution throughout the polymer layer.

Since the selected enzymes contain two copper (TYR) or two zinc atoms (ALP) chelated in their active sites, it has been possible to employ EDX analysis to prove the covalent binding of these enzymes and to map their surface distribution, as shown in Figure 5. This figure features the results of EDX analysis which confirms the presence of zinc as well as a covalent binding of ALP to electrodeposited poly-4-hydroxybenzaldehyde. Herein, it is necessary to mention that the EDX analysis was not applied on a freshly prepared biosensor, but after several measurements within the FIA mode, including additional 5 min washing with pure carbonate–bicarbonate buffer. This procedure was important to exclude non-specific adsorption of ALP molecules.

The image of zinc distribution within the poly-4-hydroxybenzaldehyde layer (see Figure 5 and inserted image) has revealed that homogeneously dispersed ALP molecules are located exclusively on the polymer coatings. In addition, it has been found that ALP molecules form aggregates that are like small islands of comparable size (>1 μm). An explanation for such structuring can be sought in the individual steps of the immobilization process, in which ALP molecules were first covalently bound to the formyl group via Schiff base formation and then other enzyme molecules fixed on the already immobilized units via glutaraldehyde and the respective cross-linking reaction.

### 3.3. Carbonyl Functionalized Polymers for Biosensing of Neurotransmitters

The electrochemical behavior of catecholamines (dopamine, noradrenaline, and adrenaline) and indoles (serotonin) in various carbonaceous electrode materials in an aqueous environment has already been investigated using cyclic voltammetry and via the corresponding electrode reactions taking place according to the ECE mechanism. This stands for the first electron transfer, whereas the proper chemical reaction is responsible for the latter transfer of electrons [29].

Generally, the above-mentioned catecholamines undergo anodic oxidation to form the respective catecholamine quinones (peak I), with the participation of two electrons and protons. These electrochemical reactions are partially reversible (peak II) because of an intramolecular cyclization that gives rise to leuco-catecholamine chromes [30]. They are further transformed via a reversible electrochemical reaction into catecholamine chromes with the participation of two electrons and protons (peaks III and IV) [31].

In acidic solutions, leuco-chromes derived from noradrenaline and adrenaline (epinephrine) are transformed via imino chromes into quinone methides, and then into indole quinones due to the presence of the hydroxy group on their alkyl chains [32]. This can be considered sufficient reason for the similarity of the cyclic voltammograms of epinephrine and serotonin (indolate), because the latter can undergo an electrochemical hydroxylation (so-called phenol pathway) [33] when forming 4,5-dihydroxytryptamine (seen as peak I), being further reversibly oxidized to tryptamine-4,5-dione with the consumption of two electrons and protons [34]. However, serotonin is mainly reversibly oxidized to serotonin quinone imine when two electrons and protons are again involved in the process (peak II) [35]. Reportedly, the formation of two different serotonin dimers has been confirmed [36].

**Figure 6:**
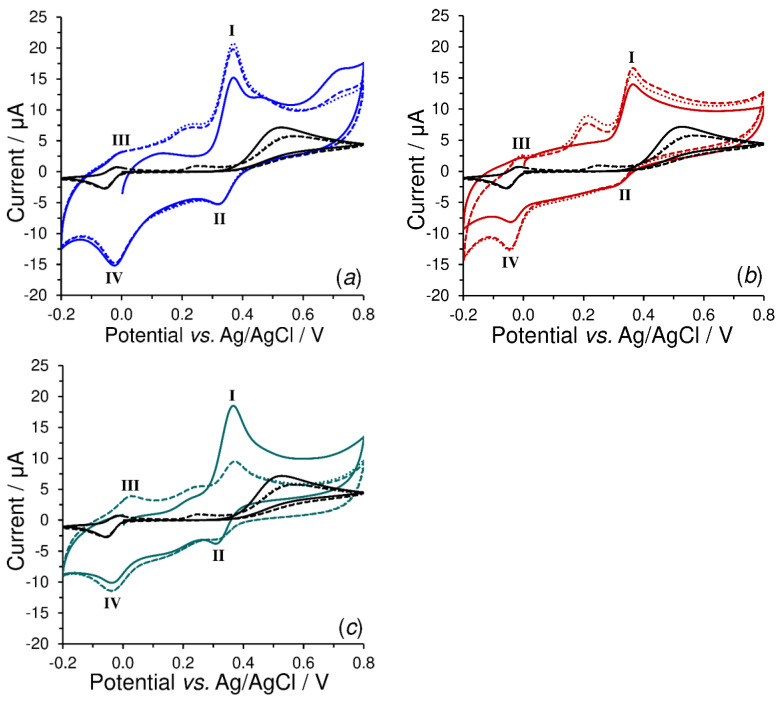
Repetitive cyclic voltammograms (1st cycle; solid, 2nd cycle; dashed and 3rd cycle; dotted curves) of 500 μmol L^−1^ (±)-epinephrine obtained at bare SPCE (black), SPCE/PV (blue; **a**), SPCE/PC (red; **b**) and SPCE/P4HB (green curves; **c**) in 0.1 mol L^−1^ AcB (pH 4.5) at *E*_step_ = 5 mV and *ν* = 10 mV s^−1^. Roman numerals denote the respective peaks (details are given in the text).

The electrochemical behavior of investigated neurotransmitters is quite different at the bare SPCE (black-colored cyclic voltammograms) than those at SPCEs covered with a thin layer of conductive polymers (blue and red cyclic voltammograms), mainly due to the presence of carbonyl groups that can interact with the primary amino groups of catecholamines, thus partially slowing down the rate of intramolecular cyclization reaction. Although it is known from the literature that the cyclization rate of various catecholamines follows a specific order: epinephrine > 3,4-dihydroxy-L-phenylalanine (DOPA) > noradrenaline > dopamine [30], the first reversible reduction peak (II) has been observed even for epinephrine, as evidenced in Figure 6.

If the voltammograms recorded in 500 μmol L^−1^ of investigated neurotransmitters (dopamine; *a*, noradrenaline; *b*, serotonin; *c*, and adrenaline; *d*) and obtained at bare SPCE (black) and SPCEs covered with all types of conductive polymers (colored curves) are compared (see Figures S1–S3), a certain conclusion can be made: their presence significantly increases the current yields of the individual electrode reactions. It seems that all the types of electrochemically deposited conductive polymers exhibit a significant electrocatalytic activity towards the (anodic) oxidation and reverse (cathodic) reduction of their corresponding catecholamine quinones (dopamine/dopamine *o*-quinone, noradrenaline/noradrenaline *o*-quinone, serotonin/tryptamine-4,5-dione and epinephrine/epinephrine *o*-quinone redox couples), which was reflected by the narrowing of these redox peaks, having increased their current yields several times, and thus deteriorating the resultant peak separation (Δ*E*_p_) from ~50 to ~160 mV.

Because the products of catalytic oxidation of neurotransmitters are identical to those produced by electrochemical oxidation with participation of two electrons and protons (dopamine *o*-quinone, noradrenaline *o*-quinone, tryptamine-4,5-dione, and epinephrine *o*-quinone), the cyclic voltammetry can be considered a suitable tool for assessing the individual amperometric transducers.

A comparison of the cyclic voltammograms shown in Figures S1–S3 indicates that SPCE/PV exhibits a high electrochemical reactivity towards dopamine, noradrenaline, and adrenaline; on the other hand, SPCE/PC exhibits only reactivity towards dopamine and noradrenaline. Thus, merely SPCE/P4HB appears to be a unique amperometric transducer suitable for the detection of tryptamine-4,5-dione in some new configurations.

**Figure 7:**
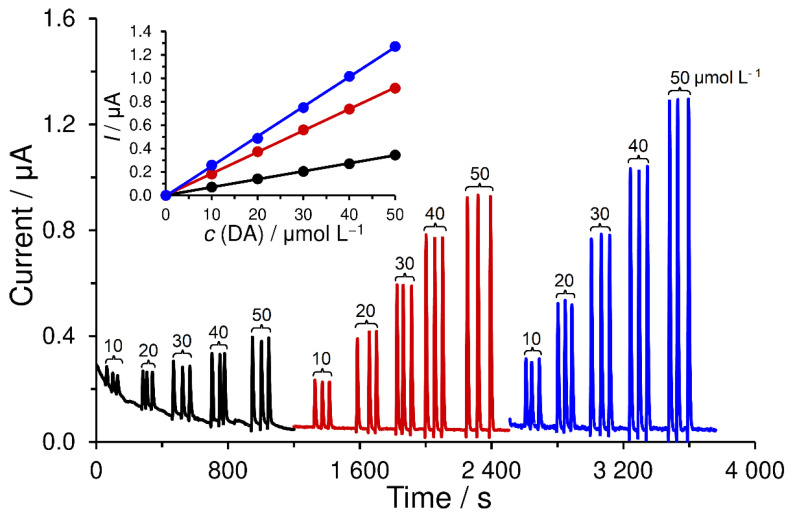
Typical amperogram of calibration measurements of dopamine (DA) obtained at bare SPCE (black), SPCE/PC (red), and SPCE/PV (blue curve) in FIA arrangement. Supporting electrolyte; 0.1 mol L^−1^ AcB (pH 4.5, not de-aerated), detection potential, +0.4 V; injection volume; 100 μL, flow rate; 1.0 mL min^−1^, and laboratory temperature; 25 °C.

As shown in Figure 7, which offers the comparison of bare SPCE (black) with SPCEs covered with poly-trans-cinnamaldehyde (red) and poly-vanillin (blue curve) in amperometric detection of dopamine (DA) within FIA, a relatively simple modification of the surface using electrodeposition caused a significant improvement in sensitivity (this can be seen in the slopes of linear regressions in the inserted image) for dopamine electrosensing, namely from 0.007 μA μmol^−1^ L for SPCE to 0.020 μA μmol^−1^ for SPCE/PC and 0.025 μA μmol^−1^ for SPCE/PV. Statistically insignificant changes (RSD > 3%) in the peak heights of the repeated analyses (triplicate injections) point to the fact that no passivation of the surface of the modified electrodes by the oxidation products of dopamine does occur because of washing away by the carrier. This is reflected in a constant current response, indicating, among others, the mechanical stability of the deposited polymers.

**Figure 8:**
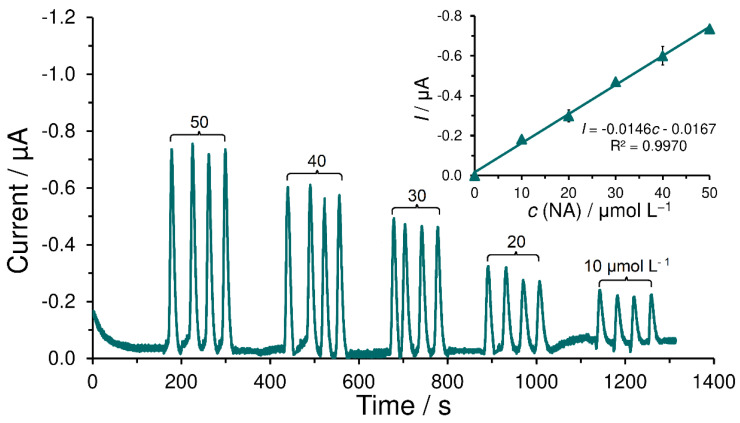
Typical amperogram of calibration measurement of noradrenaline (NA) obtained at SPCE/P4HB-TYR-GTA in an FIA arrangement. Supporting electrolyte: 0.1 mol L^−1^ PB (pH 7.0, not deaerated), detection potential; −0.2 V, injection volume; 100 μL, flow rate; 1.0 mL min^−1^, and laboratory temperature; 25 °C.

All the afore-mentioned facts suggest to us that electrodeposited carbonyl functional polymers can be used for development of sensitive voltammetric sensors or catalytic biosensors for neurotransmitter monitoring. To confirm this assumption, the mushroom tyrosinase (TYR) had been covalently bound. The resulting biosensors were subjected to model analyses. In addition to the use of derived biosensors in batch-configured measurements (see amperogram of 10 μmol L^−1^ noradrenaline (NA) obtained at SPCE/PC-TYR-GTA with the corresponding calibration curve, all in Figure S4), it seems that the polymers with an electrodeposited carbonyl functional group also represent very stable supports for enzyme molecules, even in the FIA mode, as demonstrated by the calibration measurements made with a one-week-old SPCE/P4HB-TYR-GTA biosensor, which can be seen in Figure 8. Here, it is also necessary to emphasize that these are examples of a demonstrative character, because the experiments mentioned have not been optimized with respect to maximal electroanalytical performance. Thus, the aim of this study is mainly to show what possibilities are offered by the polymers with carbonyl groups.

Nearly constant current responses for a quartet of consecutive identical injections of noradrenaline confirm the high mechanical stability of the examined biorecognition layer. Acceptable repeatability characterized by RSD < 5% was found. Everything points to the fact that the tyrosinase enzyme molecules are not washed out and the biorecognition layer is not passivated (blocked) by products coming from amperometric detection, because otherwise, in both cases, a decrease in the measured signal would occur. The simplicity and reproducibility of the preparation of such biocatalytically active layers seem to be comparable to those at polymer layers prepared by drop-casting. The mechanical stability of electrochemically deposited polymers on partially porous materials could be higher than in the case of simple adsorption.

### 3.4. Formyl Functionalized Polymers for Biosensing of Phenyl Phosphates

The electrochemical behavior of *p*-aminophenol (4-AP) and hydroquinone (HQ) had to be investigated at both bare SPCE and SPCEs covered with polymers, because they represent the products of a dephosphorylation reaction of *p*-aminophenyl phosphate (pAPP) and hydroquinone diphosphate (HQDP), respectively. To ensure optimal conditions for the catalytic activity of alkaline phosphatase (ALP), all measurements were performed in a mixed electrolyte of 0.1 mol L^−1^ carbonate-bicarbonate buffer (CBB) +0.1 mol L^−1^ KCl (pH 9).

**Figure 9:**
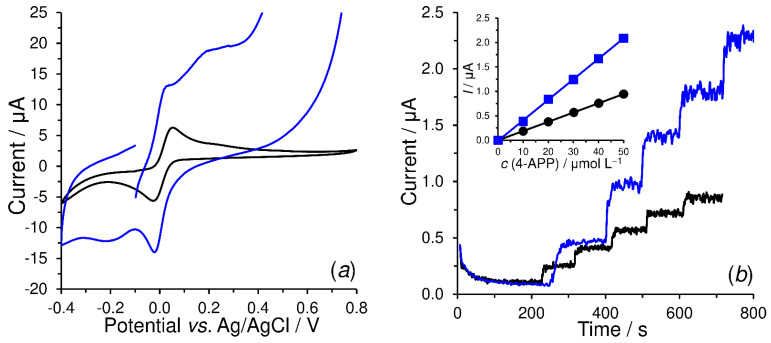
Cyclic voltammograms of 500 μmol L^−1^ *p*-aminophenol obtained at SPCE (black) and SPCE/PV (blue curve) in 0.1 mol L^−1^ CBB with 0.1 mol L^−1^ KCl (pH 9) at a scan rate of 10 mV s^−1^ (**a**). Amperograms (for batch configuration) of 10 μmol L^−1^ pAPP (five subsequent injections, *n* = 5) obtained at SPCE/ALP-GTA (black) and SPCE/PV-ALP-GTA (blue curve) in 0.1 mol L^−1^ CBB with 0.1 mol L^−1^ KCl (pH 9), when applying working potential of +0.1 V and a stirring speed of 400 rpm (**b**).

**Figure 10:**
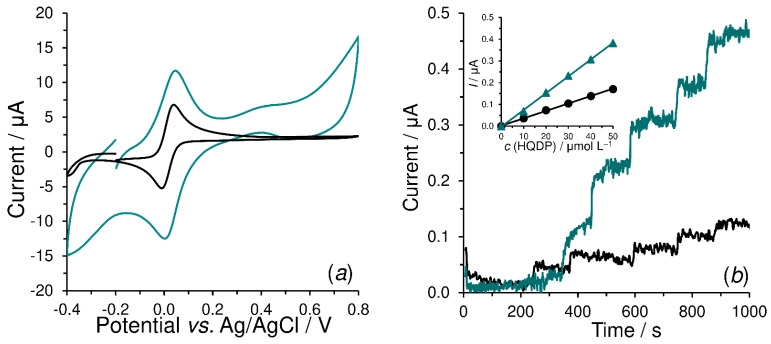
Cyclic voltammograms of 500 μmol L^−1^ hydroquinone obtained at SPCE (black) and SPCE/P4HB (deep green curve) in 0.1 mol L^−1^ CBB with 0.1 mol L^−1^ KCl (pH 9) at a scan rate of 10 mV s^−1^ (**a**). Amperograms (for batch configuration) of 10 μmol L^−1^ HQDP (five subsequent injections, *n* = 5) obtained at SPCE/ALP-GTA (black) and SPCE/P4HB-ALP-GTA (blue curve) in 0.1 mol L^−1^ CBB with 0.1 mol L^−1^ KCl content (pH 9) at working potential of +0.1 V and stirring speed of 400 rpm (**b**).

As can be expected from the literature [37,38], 4-AP and HQ provided quasi-reversible redox couples at the bare SPCE in alkaline medium (black-colored voltammograms in Figure 9a and Figure 10a), when 4-AP is oxidized to 4-quinoneimine (4-QI) and HQ to 1,4-benzoquinone (BQ), both with involvement of two electrons and protons. A similar electrochemical behavior of these substances was also observed with SPCEs covered with formyl group functionalized polymers. In the case of the electrochemical behavior of 4-AP in SPCE/PV, it was observed that the current background response of poly-vanillin itself partially overlapped the anodic peak of 4-AP at +0.020 V. Fortunately, this phenomenon did not have any significant effect on the setting of suitable working potential for subsequent amperometric detection.

In contrast to SPCE/PC, a significant increase in the current yield could be registered at SPCE/PV and SPCE/P4HB, when both these transducers provided comparable current response to each enzymatic product. Their presence was the cause of the increase in sensitivity from 0.019 to 0.042 μA mol^−1^ L, and from 0.003 to 0.008 μA mol^−1^ L in pAPP (Figure 9b), pAPP and HQDP (Figure 10b), for amperometric biosensing at +0.1 V, respectively. A higher sensitivity for pAPP than that for HQDP can be deduced from the different ALP catalytic activity towards these substrates [28,39,40]. All the facts commented in this paragraph confirm the usefulness of poly-vanillin and poly-4-hydroxybenzaldehyde in the configuration of ALP-based biosensors.

## 4. Conclusions

In this article, three monomers bearing carbonyl (*trans*-cinnamaldehyde) or formyl (vanillin and 4-hydroxybenzaldehyde) functional groups have been examined in the form of polymers electrochemically deposited onto commercially available screen-printed carbon electrode(s). When comparing the intensities of the carbonyl (C=O) stretching vibration bands of the individual polymers, it can be deduced that monomers without a hydroxyl (or thiol) group on the benzene nucleus will not represent suitable monomers for the preparation of electrochemically deposited carbonyl functional polymers. The reason is their lower electrochemical reactivity when the hydroxylation of the benzene nucleus has to occur first, which is also the case for *trans*-cinnamaldehyde. Thus, substances such as *p*-coumaraldehyde, 3-(4-hydroxy-phenyl)-propionaldehyde, and their thiolic analogues can be considered more suitable monomers for the preparation of carbonyl group functionalized polymers.

Either way, all the resulting polymeric layers have shown quite high electrochemical reactivity towards substances with amino groups (such as neurotransmitters), and thanks to that, they can be recommended as potential candidates for the construction of more sensitive voltammetric sensors, including new configurations tested in the future. Certainly, it can be predicted that analogical sensors may be widely used in food and clinical analysis thanks to their high sensitivity towards other nitrogenous organic compounds (vitamins, purines, xanthine alkaloids, indoles, etc.), making them comparable to highly sophisticated composite sensors with nanomaterials.

In addition, their high mechanical stability, even in the flow mode, makes them almost ideal supports for the preparation of biorecognition catalytic layers, which can find use in the development of analytical devices, among others. As has been experimentally proven within this study, model first-generation biosensors of this kind can be characterized by high mechanical stability in dynamic systems, including frequently used FIA arrangements.

## Data Availability

All the relevant data are only provided in the present paper.

## Supplementary Materials

The following supporting information can be downloaded at: https://www.mdpi.com/article/10.3390/s23073724/s1, Figure S1: Cyclic voltammograms of 500 μmol L^−1^ dopamine (a), noradrenaline (b), serotonin (c), and adrenaline (d) obtained at bare SPCE (black) and SPCE/PV (blue curves) in 0.1 mol L^−1^ AcB (pH 4.5) at *E*_step_ = 5 mV and *ν* = 10 mV s^−1^. Figure S2: Cyclic voltammograms of 500 μmol L^−1^ dopamine (a), noradrenaline (b), serotonin (c), and adrenaline (d) obtained at bare SPCE (black) and SPCE/PC (red curves) in 0.1 mol L^−1^ AcB (pH 4.5) at E_step_ = 5 mV and ν = 10 mV s^−1^. Figure S3: Cyclic voltammograms of 500 μmol L^−1^ dopamine (a), noradrenaline (b), serotonin (c), and adrenaline (d) obtained at bare SPCE (black) and SPCE/P4HB (green curves) in 0.1 mol L^−1^ AcB (pH 4.5) at *E*_step_ = 5 mV and *ν* = 10 mV s^−1^. Figure S4: Amperometric record (batch configuration) of 10 μmol L^−1^ noradrenaline (10 subsequent injections) obtained at SPCE/PC-TYR-GTA in 0.1 mol L^−1^ PB with 0.1 mol L^−1^ KCl content (pH 7) at working potential of −0.2 V and stirring speed of 400 rpm. The corresponding calibration curve is presented in inserted images. Scheme S1. Simplified electrochemical pathway of vanillin in aqueous environment.
