# Supplementary material for: Electrodeposited Carbonyl Functional Polymers as Suitable Supports for Preparation of the First-Generation Biosensors
Source: Sensors (Basel). 2023 Apr 4;23(7):3724. doi: 10.3390/s23073724 (PMC10098923; doi:10.3390/s23073724)
Supplement: Supplementary file 1 [file sensors-23-03724-s001.zip › sensors-2271541-supplementary.pdf]

Supplementary Materials

Electrodeposited Carbonyl Functional Polymers as Suitable Supports for Preparation of the First-Generation Biosensors

Milan Sýs, Michaela Bártová, Tomáš Mikysek and Ivan Švancara*

Department of Analytical Chemistry, Faculty of Chemical Technology, University of Pardubice, Studentská 573, 532 10 Pardubice, Czech Republic; milan.sys@upce.cz (M.S.); michaela.bartova@student.upce.cz (M.B.); tomas.mikysek@upce.cz (T.M.); ivan.svancara@upce.cz (I.S.)

* Correspondence: Correspondence: ivan.svancara@upce.cz; Tel.: +420-466-037-031

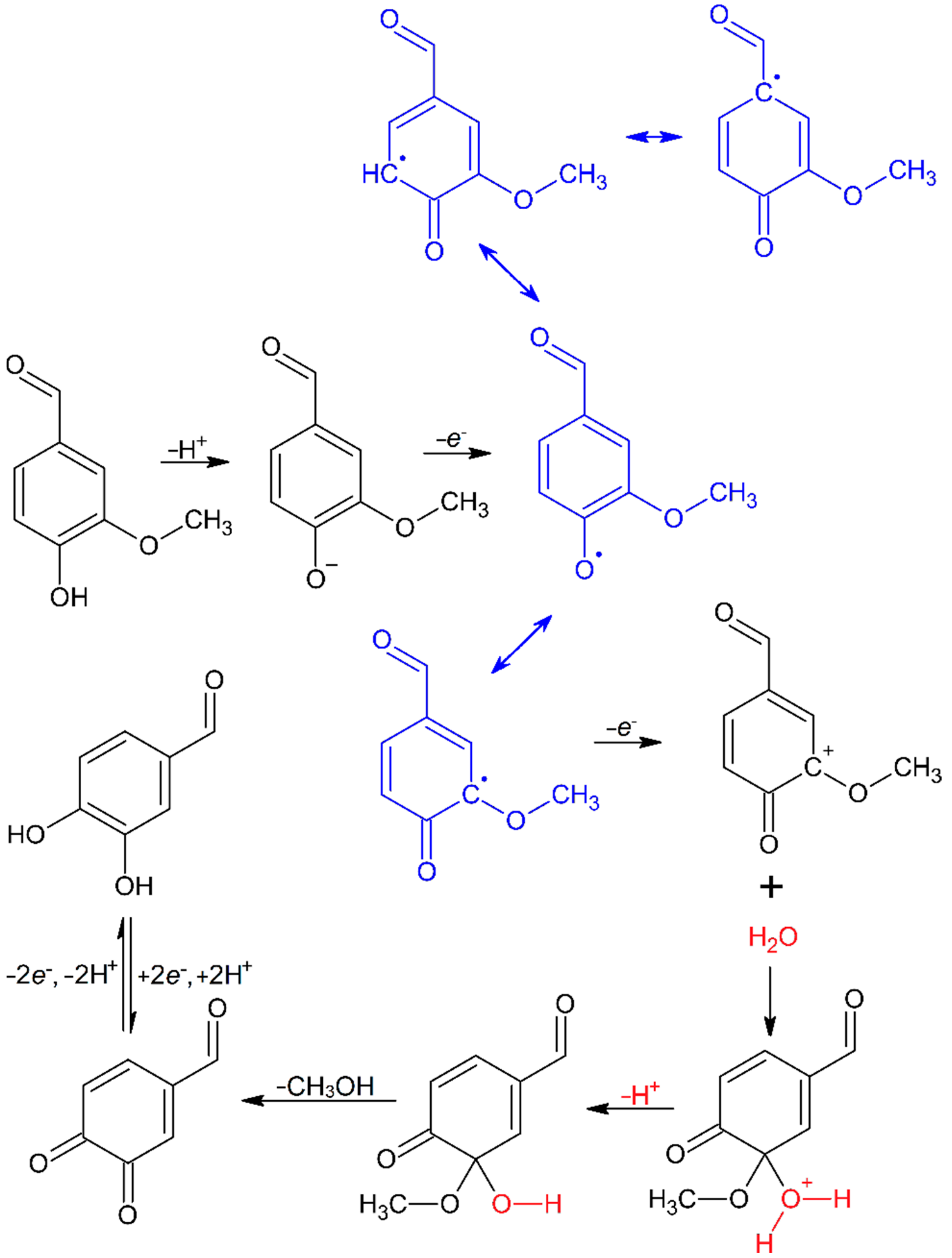

Scheme S1. Simplified electrochemical pathway of vanillin in aqueous environment.

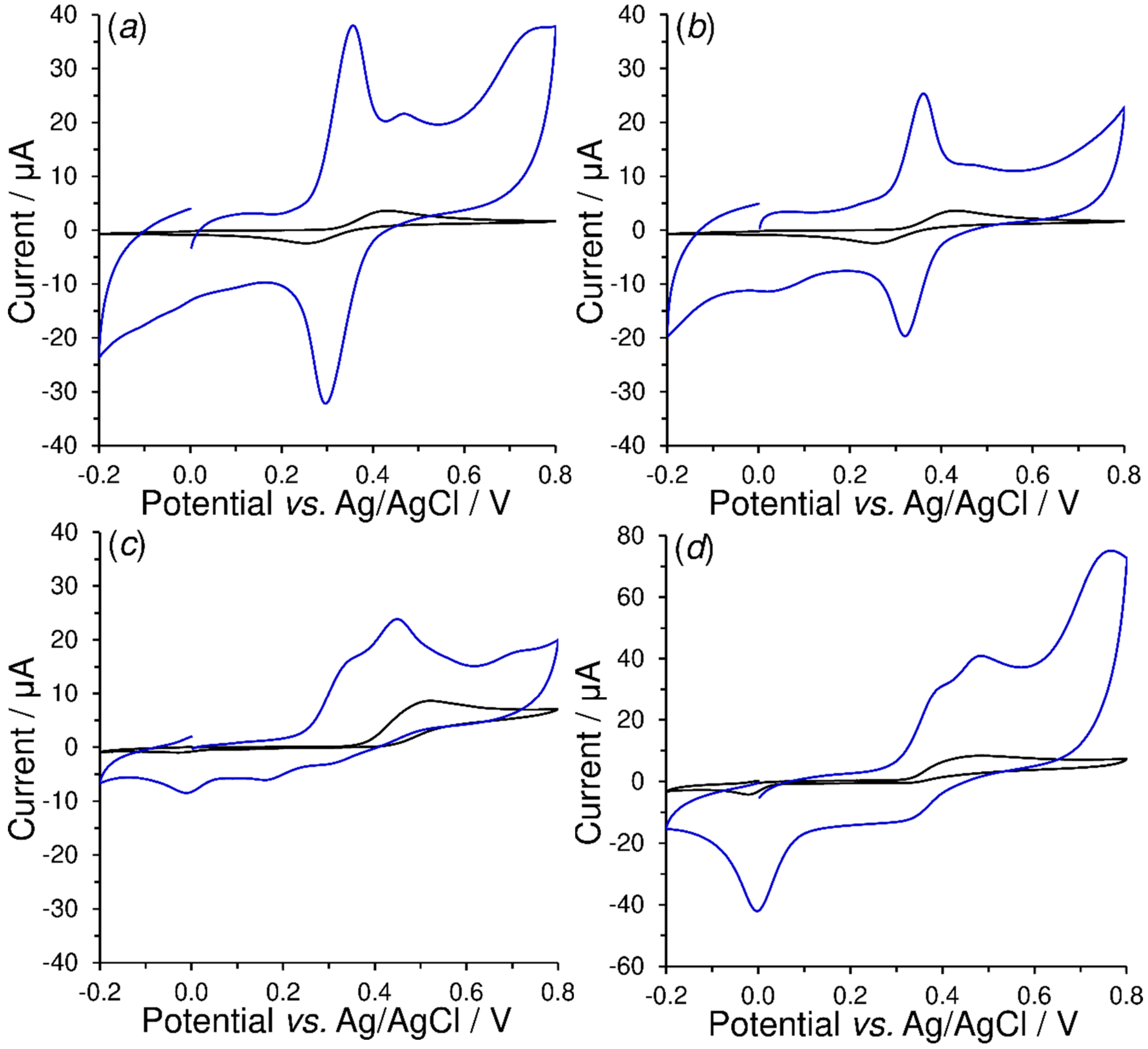

Figure S1. Cyclic voltammograms of 500 μmol L^{-1} dopamine (*a*), noradrenaline (*b*), serotonin (*c*), and adrenaline (*d*) obtained at bare SPCE (black) and SPCE/PV (blue curves) in 0.1 mol L^{-1} AcB (pH 4.5) at E_{step} = 5 mV and ν = 10 mV s^{-1}.

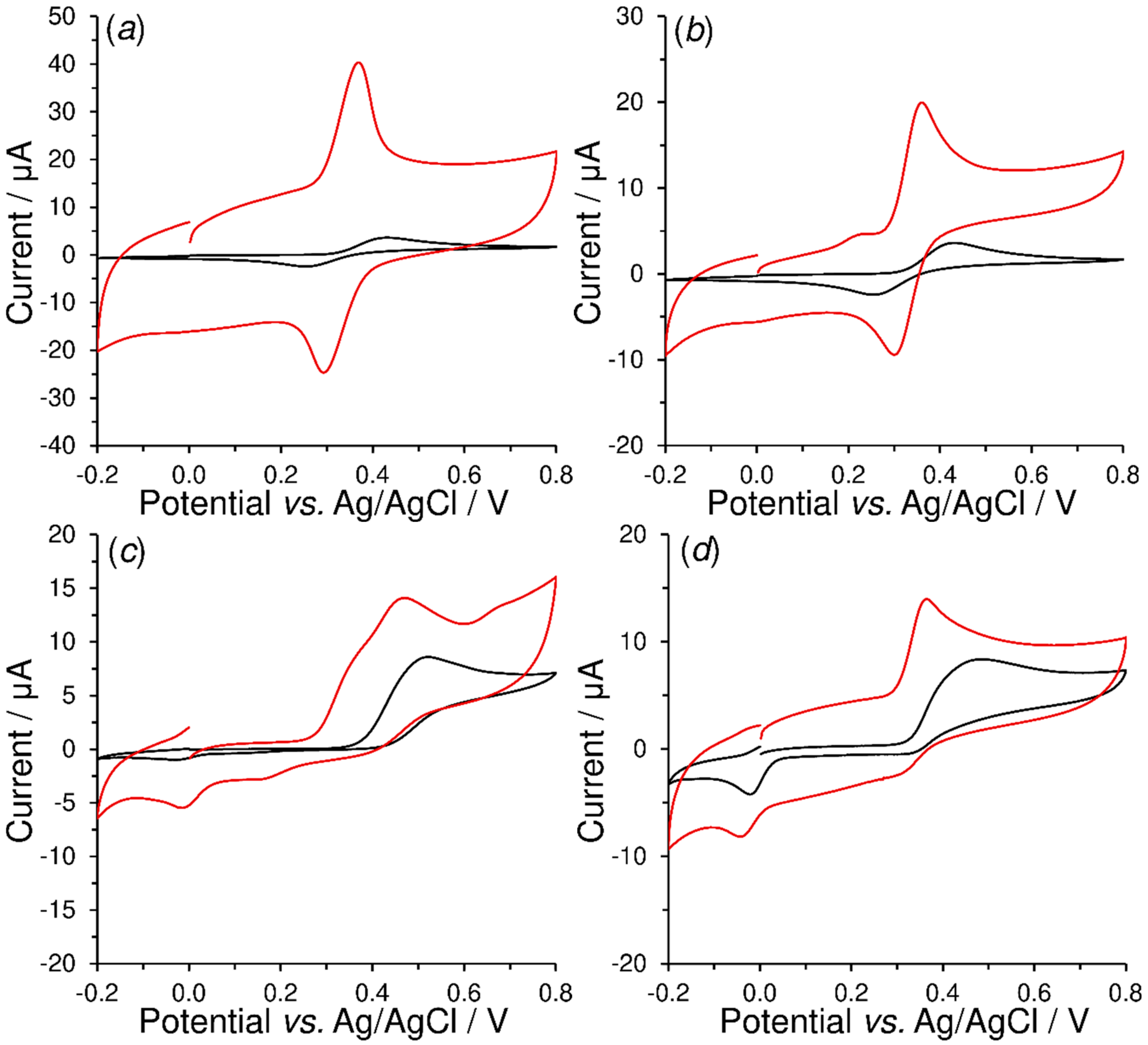

Figure S2. Cyclic voltammograms of 500 µmol L^{-1} dopamine (*a*), noradrenaline (*b*), serotonin (*c*), and adrenaline (*d*) obtained at bare SPCE (black) and SPCE/PC (red curves) in 0.1 mol L^{-1} AcB (pH 4.5) at E_{step} = 5 mV and ν = 10 mV s^{-1}.

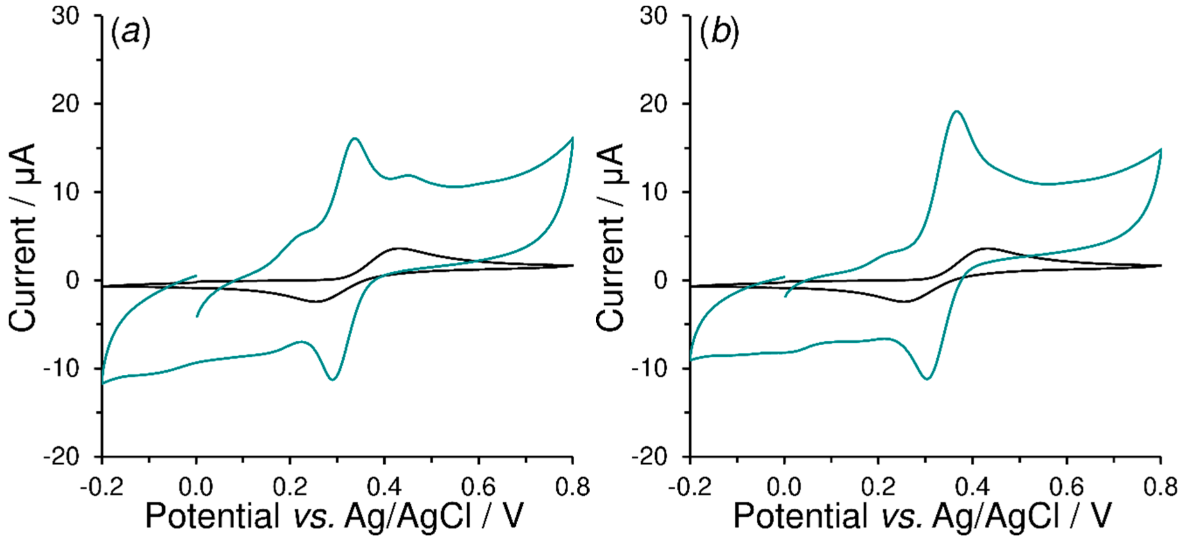

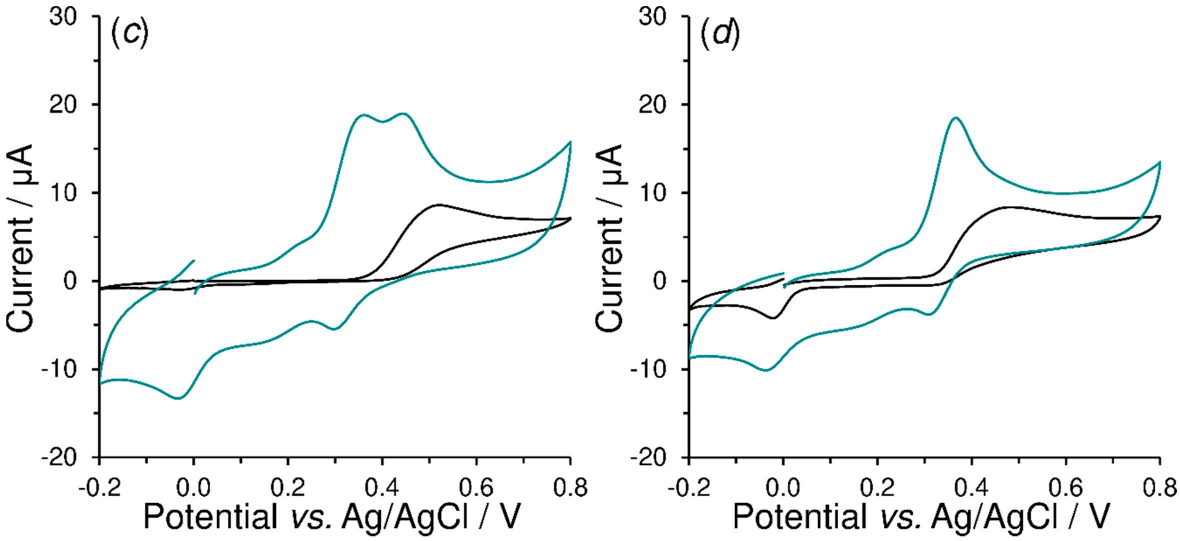

Figure S3. Cyclic voltammograms of 500 µmol L^{-1} dopamine (*a*), noradrenaline (*b*), serotonin (*c*), and adrenaline (*d*) obtained at bare SPCE (black) and SPCE/P4HB (green curves) in 0.1 mol L^{-1} AcB (pH 4.5) at E_{step} = 5 mV and ν = 10 mV s^{-1}.

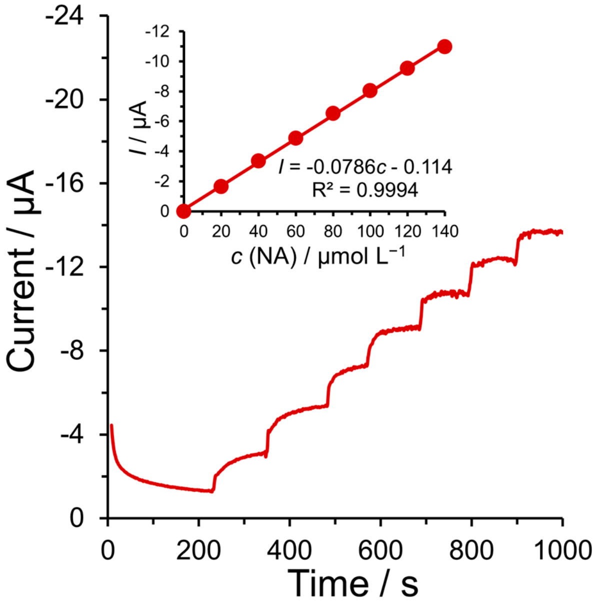

Figure S4. Amperometric record (batch configuration) of 10 µmol L^{-1} noradrenaline (10 subsequent injections) obtained at SPCE/PC-TYR-GTA in 0.1 mol L^{-1} PB with 0.1 mol L^{-1} KCl content (pH 7) at working potential of −0.2 V and stirring speed of 400 rpm. The corresponding calibration curve is presented in inserted images.
